# Emerging Roles of Long Non-coding RNAs in Chronic Neuropathic Pain

**DOI:** 10.3389/fnins.2019.01097

**Published:** 2019-10-18

**Authors:** Wei Wu, Xiaojun Ji, Yang Zhao

**Affiliations:** ^1^College of Food Science and Engineering, Qingdao Agricultural University, Qingdao, China; ^2^Department of Neurology, Affiliated Hospital of Qingdao University, Qingdao, China; ^3^Department of Anesthesiology, Affiliated Hospital to Qingdao University, Qingdao, China

**Keywords:** chronic neuropathic pain, long non-coding RNAs (lncRNAs), neuronal associated disorders, DRG neurons, glioma cells

## Abstract

Chronic neuropathic pain, a type of chronic and potentially disabling pain caused by a disease or injury of the somatosensory nervous system, spinal cord injury, or various chronic conditions, such as viral infections (e.g., post-herpetic neuralgia), autoimmune diseases, cancers, and metabolic disorders (e.g., diabetes mellitus), is one of the most intense types of chronic pain, which incurs a major socio-economic burden and is a serious public health issue, with an estimated prevalence of 7–10% in adults throughout the world. Presently, the available drug treatments (e.g., anticonvulsants acting at calcium channels, serotonin-noradrenaline reuptake inhibitors, tricyclic antidepressants, opioids, topical lidocaine, etc.) for chronic neuropathic pain patients are still rare and have disappointing efficacy, which makes it difficult to relieve the patients’ painful symptoms, and, at best, they only try to reduce the patients’ ability to tolerate pain. Long non-coding RNAs (lncRNAs), a type of transcript of more than 200 nucleotides with no protein-coding or limited capacity, were identified to be abnormally expressed in the spinal cord, dorsal root ganglion, hippocampus, and prefrontal cortex under chronic neuropathic pain conditions. Moreover, a rapidly growing body of data has clearly pointed out that nearly 40% of lncRNAs exist specifically in the nervous system. Hence, it was speculated that these dysregulated lncRNAs might participate in the occurrence, development, and progression of chronic neuropathic pain. In other words, if we deeply delve into the potential roles of lncRNAs in the pathogenesis of chronic neuropathic pain, this may open up new strategies and directions for the development of novel targeted drugs to cure this refractory disorder. In this article, we primarily review the status of chronic neuropathic pain and provide a general overview of lncRNAs, the detailed roles of lncRNAs in the nervous system and its related diseases, and the abnormal expression of lncRNAs and their potential clinical applications in chronic neuropathic pain. We hope that through the above description, readers can gain a better understanding of the emerging roles of lncRNAs in chronic neuropathic pain.

## Introduction

### Chronic Neuropathic Pain

An epidemiological survey has clearly revealed that nearly 19% of adult Europeans suffered from chronic pain of moderate to severe intensity, which not only seriously affected the physical and mental quality of their social and working lives but also represents a significant public health issue that can be costly to the healthcare system (Rapo-Pylkko et al., 2017). Among all the different types of chronic pain, neuropathic pain is relatively common, but difficult to treat and manage (Baron, 2006, 2009). Moreover, neuropathic pain occurs in approximately 7–10% of the general population; therefore, neuropathic pain remains one of the most serious public health problems (van Hecke et al., 2014). Traditionally, the International Association for the Study of Pain (IASP) proposed a clinical description for neuropathic pain, which is defined as “pain initiated or caused by a primary lesion or dysfunction in the nervous system” (Scholz et al., 2019). However, this definition is broad, covering over 100 conditions, and has been controversial in recent years (National Institute of Neurological Disorders Stroke rt PASSG, 2005; Meacham et al., 2017). The main reason is that many scholars believe that the meaning of the term “dysfunction” is still relatively vague (Baron, 2009). Furthermore, it does not distinguish well between neuropathic pain and nociceptive pain (Vranken, 2012). Recently, a new precise definition of neuropathic pain has been put forward to avoid the above shortcomings, and to further beneficially support clinical and research purposes (Lauria et al., 2012; Treede et al., 2019). The revised definition is that “pain arose as a direct consequence of a lesion or disease affecting the somatosensory system,” which definitely indicates that the peripheral or central somatosensory system must be involved (Scholz et al., 2019; Treede et al., 2019). According to the underlying etiology and the anatomical location of the specific lesion, clinical neuropathic pain is haply divided into four main types, namely, painful peripheral neuropathies, central pain syndromes, complex painful neuropathic disorders, and mixed-pain syndromes (Baron, 2009; Nijs et al., 2016). Painful peripheral neuropathies are caused by ischemic, traumatic, inflammatory, infectious, metabolic, or compressive damage to the peripheral nervous system (PNS), such as polyneuritis, phantom limb pain, diabetic peripheral neuralgia, post-herpetic neuralgia, etc. (Beran, 2015; Watson and Dyck, 2015). Central pain syndromes result from the dysfunction of or damage to the central nervous system (CNS) pain conduction pathway, mainly involving cerebrovascular disease, spinal cord trauma, etc. (Petzke, 2010). Complex painful neuropathic disorders are also called “complex regional pain syndromes (CRPS),” which may result in trauma and affect the limbs (Baron and Wasner, 2001; Borchers and Gershwin, 2014). In addition, mixed-pain syndromes are the combination of nociceptive and neuropathic pain (Baron, 2009; Picelli et al., 2016).

Generally, patients with neuropathic pain may show distinct negative and positive sensory symptoms or signs in the nerve distribution area (Baron, 2006). Firstly, the negative sensory symptoms predominately contain hypoesthesia, pallhypoesthesia, hypoalgesia, thermohypoesthesia, etc. (Baron, 2006; Shaygan et al., 2014). Secondly, the positive sensory symptoms primarily include paresthesia, paroxysmal pain, superficial pain, and evoked pain. This would stimulate induced hypersensitivity and pain, such as light-pressure, gentle static pressure, and sharp pinprick (Baron, 2006; Soler et al., 2017). However, in a clinical setting, patients often were diagnosed as hyperesthesias that are mainly referred to as paresthesia and hyperalgesia (Zimmermann, 2001). The two types of hyperesthesia need to be clearly distinguished. Paresthesia (also known as mechanodynamic painful paresthesia) is defined as the non-noxious stimuli (e.g., light touch, warm, cool), and normally would not cause painful sensation in the normal nerve areas, but it could cause strongly painful sensation in the damaged nerve areas (Baron, 2009; Arle et al., 2016). Nevertheless, hyperalgesia is defined as an increased pain sensitivity or painful summation to mildly noxious stimuli (Baron, 2009; Jensen and Finnerup, 2014). To date, pharmacotherapy is the main therapeutic strategy in neuropathic pain patients. However, these drugs and agents lack specificity and efficiency, and could reduce the pain to a more tolerable level (Teixeira, 2009; Helfert et al., 2015). Thus, it is urgently needed to further explore the potential molecular mechanisms of the neuropathic pain development, which may provide a rational solution for clinical treatment and novel drug discovery in neuropathic pain.

### Long Non-coding RNAs (lncRNAs)

With the development of larger-scale sequencing techniques and bioinformatics methods, the Encyclopedia of DNA Elements (ENCODE) project, the most comprehensive effort yet for surveying transcription in human cells, has clearly uncovered that only approximately 2% of the whole human genome encodes functional protein-coding genes, while the remaining genome is actively transcribed into a diversity of RNAs that were defined as non-coding RNAs (ncRNAs) and were previously believed to be non-functional “dark matter” (Qu and Fang, 2013). However, an accumulating body of evidence in recent years reported that, similar to protein-coding genes, ncRNAs are also involved in a wide variety of biological processes, such as cell proliferation, cellular structure integrity, cellular development, differentiation, stem cell pluripotency, signaling transductions, genomic imprinting, the maintenance of genomic integrity, reprogramming, heat shock response, etc. (Nicolas, 2017; Sherstyuk et al., 2018). Therefore, it was no surprise that abnormal expression profiles of ncRNAs were implicated in the initiation, development, and progression processes of many human diseases, including cardiovascular syndromes, diabetes, autoimmune diseases, nervous system disorders, cancers, and so on (Huang et al., 2013). However, among all classifications of ncRNAs, lncRNAs, a heterogeneous group of transcripts that are greater than 200 nucleotides (nt) in length, account for more than 80% of ncRNAs (Jarroux et al., 2017). Because lncRNAs occupy the largest class of ncRNAs and represent the most prevalent and functionally diverse class of ncRNAs, they attract interest in the scientific community in the past decades (Roberts et al., 2014).

**FIGURE 1:**
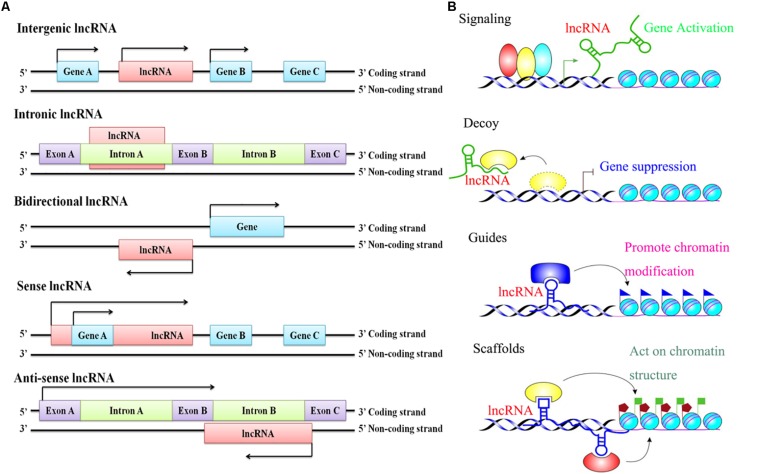
The classification and functional mechanisms of lncRNAs. **(A)** Classification of lncRNAs according to the relative proximity to the nearest protein-coding transcripts; **(B)** Functions of lncRNAs in regulating target genes.

Since Brannan and his colleagues reported the first lncRNA H19 in 1990, thousands of lncRNAs have been identified in a large diversity of species, including mammalian animals, plants, yeast, prokaryotes, and even viruses, by a large number of scientists (Jarroux et al., 2017). It is reported that the lncRNA is bidirectional transcribed, depending on their proximity to the nearest protein-coding transcripts. It can be grouped into five main categories: sense lncRNAs (lncRNAs initiate inside or 5’ of a protein-coding gene, then are transcribed in the same direction as protein-coding genes, and ultimately overlap at least one protein-coding exon), antisense lncRNAs (lncRNAs initiate inside or 3’ of a protein-coding gene, then are transcribed in the opposite direction of protein-coding genes, and ultimately overlap at least one protein-coding exon), bidirectional lncRNAs (the expression of the lncRNA and its neighboring protein-coding genes on the opposite strand is initiated in close genomic proximity), intronic lncRNAs (the entire sequence of the lncRNA falls within the intron of a protein-coding gene), and intergenic lncRNAs (also termed large intervening ncRNAs or lincRNAs, in which the entire sequence of the lncRNA falls between two protein-coding genes as a distinct unit) (Figure 1A; Ma et al., 2013; St Laurent et al., 2015). In addition, an increasing number of studies have found that lncRNAs play versatile roles in many aspects of gene regulation through four functional mechanisms: signaling and acting as a decoy, guide, and scaffolding molecule (Figure 1B; Akhade et al., 2017; Delas and Hannon, 2017). First, as a signaling molecule, lncRNAs are usually transcribed in a specific time and space, and their transcripts can integrate developmental cues, interpret cell status, or respond to multiple stimuli and further regulate the expression of other genes. Therefore, lncRNAs are seen as an important marker in a biological event (Wang and Chang, 2011). Second, as a decoy molecule, lncRNA can block the binding of transcriptional repressors to their homologous target gene promoters, as well as protein molecules, chromatin modifications, etc., and thereby lncRNAs could ultimately inhibit their functions (Ma et al., 2012). Third, as a guide molecule, lncRNAs are able to directly bind to the protein molecules and then guide the protein complexes containing the above-described protein-bound molecules to be precisely localized on a specific target (Wang and Chang, 2011). On the other hand, lncRNA can also recruit more transcription-related and epigenetic-related regulatory factors widely and further guide their localizations (Goff and Rinn, 2015). Finally, as a scaffold molecule, lncRNAs have many different structural domains that can combine different effector molecules to achieve the assembly of macromolecules at the same time. In fact, lncRNA is a complex assembly center platform, which can recruit many related molecules together to further activate or inhibit the expression of other genes (Bonasio et al., 2010; Ma et al., 2012). It is extremely important for the transmission of many biological signals, intermolecular interactions, and the precise regulation of the specificity and dynamics of the signal itself (Wang and Chang, 2011; Moran et al., 2012). Hence, based on the multiple functions of lncRNAs, researchers have gradually shifted their focus of research over the last few decades from previous studies on protein-coding gene-induced diseases to studies on lncRNA-induced diseases (Moran et al., 2012; Delas and Hannon, 2017).

## The Roles of lncRNAs in the Nervous System and Its Related Diseases

The human nervous system is the most highly evolved and sophisticated biological system that plays a leading role in the regulation of physiological and functional activities in the body (Herculano-Houzel, 2009; Herculano-Houzel et al., 2014). Generally, the functions and physiological processes of different organs and systems in the human body are not carried out in isolation, but they are closely connected, interacting and cooperating under the direct or indirect control of the nervous system, which makes the human body become a complete and unified organism to achieve and maintain normal life activities when the human body encounters interoceptive and environmental stimuli (Herculano-Houzel, 2012; Kohl and Dulac, 2018). It is well-known that the human nervous system is mainly composed of nerve tissues that contain two basic types of cells, neurons and glial cells, and is divided into two major parts, the CNS and the PNS (Garner and Mayford, 2012; Zimmerman et al., 2017). The CNS, containing the brain and the spinal cord, is where we receive sensory information, generate thoughts and emotions, and store memories (Kaucka and Adameyko, 2014; Engelhardt et al., 2017). The perfect presentation of CNS and PNS functions predominately depends on the dynamic neural networks organized by neuronal and glial cells (Guarino et al., 2017). However, the development of these cells requires a precise spatiotemporal regulation of stem/progenitor cell proliferation and differentiation at various levels, such as epigenetic, transcriptional, or post-transcriptional processes, and then forms appropriate connections with each other (Qureshi and Mehler, 2010). In recent years, emerging data have reported that an increasing number of lncRNAs were found to play vital roles in mediating the developmental complexity of the nervous system, and these abundant lncRNAs are precisely, dynamically, and specifically expressed in the brain via temporal and spatial expression patterns (Qureshi and Mehler, 2013). In fact, it is not difficult to understand that the development of the nervous system is actually a complex neural network formed by the tight gene regulation of its constituent cells (Garner and Mayford, 2012). Nevertheless, based on the explanation of the functions of lncRNAs in the above, it can be known that the regulatory mechanisms of lncRNAs may participate in the entire process of nervous system development (Wu et al., 2013). Therefore, not surprisingly, the dysregulation of or mutations in lncRNA gene loci might directly affect nervous system development, function, maintenance, and differentiation and be intimately associated with the molecular pathophysiology of a broad array of the most devastating neurological diseases, such as glioma, neuropathic pain, autism spectrum disorder (ASD), Alzheimer’s disease (AD), Parkinson’s disease (PD), Huntington’s disease (HD), Asperger’s syndrome, schizophrenia, developmental delay, amyotrophic lateral sclerosis (ALS), depression, autism, and others (Riva et al., 2016; Wan et al., 2017).

First, we explored the roles of lncRNA in neuronal development (Wu et al., 2013; Ramos et al., 2016). Several lines of evidence have demonstrated that the profiles of lncRNA expression present a dynamic change during the process of neuronal development (Rani et al., 2016). For example, Mercer et al. discovered that 1328 lncRNAs were identified in the mouse, whereas among these lncRNAs, 849 lncRNAs were located in the mouse brain with 623 lncRNAs exhibiting selective profiles for specific regions (e.g., olfactory bulb, hippocampus, cortex, or cerebellum), cell types (e.g., neurons, glia cells), and subcellular compartments (e.g., dendrite, axon, Nissl body, soma, etc.), which suggested that these lncRNAs might be involved in regulating specific processes through cell- type-, subcellular- compartment-, and developmental-stage-specific manners (Bussotti et al., 2016). Amaral et al. revealed that the lncRNA Sox2OT was expressed in regions of constitutive adult neurogenesis and mediated the expression of the Sox2 gene, which is a key transcription factor (TF) that is responsible for the neural induction and maintenance of neural stem and progenitor cells, indicating that the lncRNA Sox2OT might participate in neural cell fate decisions (Amaral et al., 2009). Other studies related to lncRNAs in neuronal development are shown in Table 1. Throughout the study of these lncRNAs in neurodevelopment, it is known that the methods of exploring the physiological functions of lncRNAs are basically the same. Researchers often use qRT-PCR to detect changes in the expression of lncRNAs in blood samples from patients with neurological diseases, in cell model samples, or in animal model samples. Moreover, a dual-luciferase reporter assay was applied to verify the target interaction between lncRNAs and their downstream target genes. In addition, CCK8, colony formation assay, and flow cytometry were utilized to assess the influences of lncRNA on neuronal development.

**TABLE 1:** lncRNAs’ roles in neuronal development.

**lncRNAs**	**Location**	**Expression**	**Samples**	**Roles of lncRNAs in**	**References**
				**neuronal development**	
BC1 (Brain cytoplasmic RNA 1)	Chromosome 7	Down-regulated	Mice	Changes behavioral phenotypes including reduced exploration and increased anxiety	Lewejohann et al., 2004
BRN1B (also named Pantr2, POU domain, class 3, transcription factor 3 adjacent non-coding transcript 2)	Chromosome 1	Down-regulated	Developing mouse pups	Controls differentiation of delaminating neural progenitor cells	Sauvageau et al., 2013
DALI (DNMT1 associated lincRNA)	Chromosome 1	Up-regulated	Neuroblastoma cells	Drives the expression of an essential neuronal differentiation gene expression program	Chalei et al., 2014
EVF2 (also termed DLX6-AS1, DLX6 antisense RNA 1)	Chromosome 7	Down-regulated	Mice	Disrupts the excitatory to inhibitory neuron balance in the post-natal hippocampus and dentate gyrus	Bond et al., 2009
GOMAFU (also called MIAT, myocardial infarction associated transcript)	Chromosome 5	Down-regulated	Mice	Regulates splicing of several neuronal genes and increases amacrine cell and Muller glia differentiation	Rapicavoli et al., 2010; Briggs et al., 2015
KCNA2-AS (Potassium voltage-gated channel subfamily A member 2 antisense RNA)	–	Up-regulated	The PNS of rats	Implicate in the control of neuronal plasticity	Briggs et al., 2015
Nkx2.2AS (NKX2-2 antisense RNA)	20p11.22	Up-regulated	Neural stem cells (NSCs)	Enhances oligodendrocytic differentiation	Tochitani and Hayashizaki, 2008
NTAB (Nitrilotriacetate monooxygenase component B)	–	Up-regulated	Developing and adult rat brain	Involve regulation of RNA transport or translation in neuronal processes	French et al., 2001
PAUPAR (PAX6 upstream antisense RNA)	11p13	Up-regulated	Neuroblastoma cells	Regulates a transcriptional program that influences the cell-cycle profile and differentiation	Vance et al., 2014
PNKY (A neural-specific lncRNA)	Chromosome 6	Down-regulated	Dividing NSCs of mouse and human brain	Controls the balance between self-renewal and neuronal differentiation	Ramos et al., 2015
RMST (Rhabdomyosarcoma 2 associated transcript)	12q23.1; 12q21	Up-regulated	Human brain, Human embryonic stem cells (hESCs)	Required for neural differentiation	Ng et al., 2013
SIX3OS (SIX homeobox 3, opposite strand 1)	Chromosome 17	Up-regulated	Mice	Controls the specification of photoreceptors, bipolar cells, and Muller glia and regulates retinal development	Rapicavoli et al., 2011
TUG1 (Taurine up-regulated 1)	Chromosome 11	Up-regulated	Mice	Involved in retinal development	Young et al., 2005
TUNA (Tcl1 upstream neuron-associated lincRNA)	Chromosome 12	Up-regulated	Mice embryonic stem cells (mESCs)	Controls pluripotency and neural lineage commitment	Lin et al., 2014

Second, we continue to investigate the effects of lncRNAs on neurological diseases (Riva et al., 2016). For instance, over-expressed lncRNA HOXA transcript antisense RNA, myeloid-specific 1 (HOTAIRM1), facilitates tumor growth and invasion through up-regulating HOXA1 and sequestering G9a/EZH2/Dnmts away from the HOXA1 gene in glioblastoma multiforme (Li Q. et al., 2018). LncRNA small nucleolar RNA host gene 12 (SNHG12) accelerates angiogenesis following ischemic stroke via a miR-150/VEGF pathway, which further clarifies the mechanism of angiogenesis after ischemic stroke and provides a target for the treatment of this disease (Zhao M. et al., 2018). The imbalance of lncRNA AK127244 might have a particular influence on language development and behavior or mood in neuropsychiatric disorders (Duong et al., 2015). Nuclear factor-k-gene binding (NF-kB) interacting lncRNA (NKILA) promotes the endoplasmic reticulum stress/autophagy pathway and suppresses the NF-kB pathway after intracerebral hemorrhage (Jia et al., 2018). Other studies linked to lncRNAs in neuronal-associated disorders are detailed in Table 2.

**TABLE 2:** lncRNAs’ roles in neuronal associated disorders.

**lncRNAs**	**Location**	**Expression**	**Samples**	**Neuronal associated disorders**	**Roles of lncRNAs in neuronal associated disorders**	**References**
ANRIL (Antisense non-coding RNA in the INK4 locus)	9p21.3	Up-regulated	Patients	Intracranial aneurysms	ANRIL may become a molecular marker of intracranial aneurysms in the future	Che, 2017
AK042766	–	Up-regulated	Mice	Restless Legs Syndrome (RLS)	AK042766 may regulate the expression of the Meis1 gene during the pathogenesis of Restless Legs Syndrome (RLS)	Ponjavic et al., 2009
BC200 (also termed BCYRN1, brain cytoplasmic RNA 1)	2p21	Up-regulated	Patients	AD	BC200 is involved in the synaptic and neural network dysfunction that is found in both early and later stages of AD	Li H. et al., 2018
BACE1-AS (Beta-secretase 1 antisense RNA)	11q23.3	Down-regulated	Patients, mice	AD	BACE1-AS drives overproduction of toxic AB-42 peptides, which then feedback to further induce BACE1-AS overexpression, accelerating amyloid accumulation and finally leading to the generation of AD	Faghihi et al., 2008
DISC2 (Disrupted in schizophrenia 2)	1q42.2	Down-regulated	Patients	Neuropsychiatric disorders	DISC2 has been implicated in the development of neuropsychiatric disorders, such as autism spectrum disorder	Williams et al., 2009
H19 (Human homolog 19)	11p15.5	Up-regulated	Patients, human glioma cell lines	CNS tumors	H19 deregulation may be relevant for CNS tumors, such as glioma	Muller et al., 2000
GOMAFU	Chromosome 5	Down-regulated	Mice, human pluripotent-cell-derived neurons	Schizophrenia	GOMAFU may be involved in driving this aberrant splicing of DISC1 and ERRB4 in schizophrenia	Barry, 2014; Briggs et al., 2015
KCNA2-AS	–	Up-regulated	Rat	Neuropathic pain	KCNA2-AS appears to be a key driver of neuropathic pain symptoms	Briggs et al., 2015; Li et al., 2019
M21981	–	Up-regulated	Mice	MS	M21981 are involved in abnormal CD8+ T-cell differentiation and activation in the pathophysiology of MS	Hafler et al., 2007; Qureshi et al., 2010
MEG3 (Maternally expressed gene 3)	14q32.2	Up-regulated	Glioma cells U251	Glioma	MEG3 inhibits proliferation and migration but induces autophagy by regulation of Sirt7 and PI3K/AKT/mTOR pathway in glioma cells	Xu et al., 2018
MSNP1AS (Moesin pseudogene 1 antisense RNA)	5p14.1	Up-regulated	Patients	ASD	MSNP1AS may regulate MSN protein by binding to and stabilizing MSN mRNA, and that this mechanism may causally connect SNP variants in the MSNP1AS locus to ASD pathogenesis	Kerin et al., 2012; DeWitt et al., 2016
REST/CoREST-regulated lncRNAs	–	Down-regulated	Patients	HD	The potential disruption of REST-regulated lncRNA expression in HD may lead to additional disturbances in lncRNA-mediated chromatin and transcriptional regulatory processes through a feed-forward mechanism	Johnson, 2012
UCH1LAS (Ubiquitin C-terminal hydrolase L1 antisense RNA)	4p14	Up-regulated	Patients	PD	UCH1LAS involves in regulating pathways related to the development of PD	Kraus et al., 2017
XCI (X chromosome inactivation)	Chromosome X	Up-regulated	Patients	CNS tumors	Perturbations in XCI expression are associated with CNS tumors	Qureshi et al., 2010

## The Abnormal Expression of lncRNAs and Their Potential Clinical Applications in Chronic Neuropathic Pain

**FIGURE 2:**
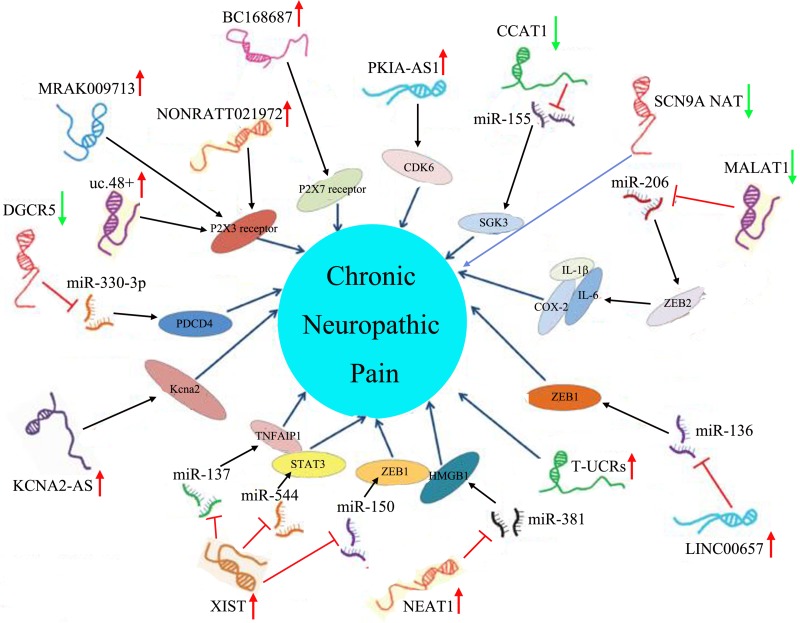
Aberrant lncRNA expression and their downstream molecular pathways in chronic neuropathic pain. The red arrow represented an up-regulated trend of lncRNAs, and the green arrow represented a down-regulated trend of lncRNAs.

Chronic neuropathic pain generally resulting from injury to or disease of the nervous system has always been very difficult in clinical treatment, mainly because almost all patients with chronic neuropathic pain are notoriously resistant to the actions of currently available analgesics, such as non-steroidal anti-inflammatory drugs and opioids, which could exert effective therapeutic roles in nociceptive pain (Jackson, 2006). Pharmacological (e.g., tricyclic antidepressants, anticonvulsants, selective serotonin norepinephrine reuptake inhibitors, and topical lidocaine), non-pharmacological (e.g., behavioral, cognitive, integrative, and physical approaches), and interventional (e.g., neuroablative and neuromodulation techniques) therapies are currently used in the treatment of chronic neuropathic pain. These treatments cannot completely control the symptoms of patients with chronic neuropathic pain, which may be associated with the diversity of pathophysiological situations, different pain etiologies, genetic predispositions, etc. (Gilron et al., 2015). Thus, chronic neuropathic pain remains a distressing and debilitating disease, there is still no panacea for the treatment of chronic neuropathic pain in the clinic, and it is imperative to further develop chronic neuropathic pain drugs (Jensen and Finnerup, 2007; Xu et al., 2016). In recent years, a large number of studies have revealed that many prominent and well-characterized mechanisms were observed in chronic neuropathic pain, including (1) ongoing ectopic activity in peripheral nerves, which resulted in excitation–inhibition imbalance; (2) the aberrant production and release of anti-inflammatory or proinflammatory cytokines at the site of injury, which triggers peripheral sensitization or central sensitization; (3) the impairment of endogenous inhibitory mechanisms of nociception sensitivity-induced microglial sensitivity after nerve injury, which enhanced the post-injury pain sensitivity; and (4) microglial activation after nerve injury, which contributed to the pathogenesis of pain hypersensitivity (Gilron et al., 2015). Therefore, in the full understanding of the pathogenesis of chronic neuropathic pain, we can develop corresponding new therapeutic drugs for chronic neuropathic pain patients according to their relevant mechanisms (Gilron et al., 2006; Vranken, 2012). Furthermore, most extensive evidence has definitely pointed out that there were many dysregulated lncRNAs expressed in damaged nerves, primary sensory dorsal root ganglion neurons, spinal cord dorsal roots, the prefrontal cortex, the post-synaptic dorsal horn, and even in higher-order neurons up to the cortical level after peripheral nerve injury; therefore, it was speculated that these dysregulated lncRNAs might be involved in the pathogenesis of chronic neuropathic pain (Li et al., 2019). Furthermore, this speculation was also demonstrated by a large number of researchers. For instance, Liu et al. constructed a mouse model of spared nerve injury-induced neuropathic pain and found, by microarray technology, that there were 22,213 abnormally expressed lncRNAs, which might be involved in the activities of cytokines (IL-17A and IL-17F) and chemokines (CCL-2, CCL-5, and CCL-7), in the spinal cord, suggesting that differentially expressed lncRNAs played a pivotal role in the progression of neuropathic pain (Liu Z. et al., 2017). Additionally, Jiang et al. identified 511 differentially expressed (>2-fold change) lncRNAs (366 up-regulated and 145 down-regulated) in the spinal cord of mice following spinal nerve ligation-induced neuropathic pain, and these lncRNAs might be related to Toll-like receptor signaling, cytokine–cytokine receptor interaction, and peroxisome proliferator-activated receptor signaling pathways, which indicated that abnormally expressed lncRNAs might be implicated in the pathogenesis of chronic neuropathic pain (Jiang et al., 2015). With the deepening of lncRNA research, scientists are no longer limited to the study of lncRNA expression profiles of chronic neuropathic pain, and they have begun to specifically explore the biological function of these differentially expressed lncRNAs in chronic neuropathic pain (Wu et al., 2019). Thus, the amassing literature referring to lncRNAs in chronic neuropathic pain is presented in detail in Table 3 and Figure 2.

**TABLE 3:** Comprehensive analysis of aberrant lncRNA expression profiles in chronic neuropathic-related conditions.

**lncRNAs**	**Expression levels**	**Localization of lncRNAs expression**	**Samples**	**Potential regulatory mechanism of dysregulated lncRNAs**	**References**
BC168687	Up-regulated	DRG neurons	Streptozotocin-induced diabetic rats	lncRNA BC168687 may participate in the pathogenesis of diabetic neuropathic pain mediated by P2 × 7 receptor.	Liu C. et al., 2017
CCAT1 (Colon cancer-associated transcript-1)	Down-regulated	DRG neurons, spinal dorsal horn, hippocampus and anterior cingulated cortex	Bilateral sciatic nerve CCI rats	lncRNA CCAT1 overexpression could alleviate the pain thresholds and promote the expression of serum and glucocorticoid regulated protein kinase 3 (SGK3) through sponging miR-155.	Dou et al., 2017
DGCR5 (DiGeorge syndrome critical region gene 5)	Down-regulated	DRG neurons	CCI rats	DGCR5 overexpression was able to alleviate neuropathic pain development including mechanical and thermal hyperalgesia through sponging miR-330-3p and regulating PDCD4 in CCI rat models.	Peng et al., 2019
KCNA2-AS (Potassium voltage-gated channel subfamily A member 2 antisense RNA)	Up-regulated	DRG neurons	Rat, mouse, monkey and human	Overexpression of KCNA2-AS down-regulated Kcna2, reduced total voltage-gated potassium current, increased excitability in DRG neurons, and produced neuropathic pain symptoms, but blocking KCNA2-AS reversed nerve injury-induced down-regulation of DRG Kcna2 and attenuated development and maintenance of neuropathic pain.	Li et al., 2019
LINC00657	Up-regulated	DRG neurons	CCI rats	LINC00657 suppressed neuropathic pain-related symptoms, such as mechanical and thermal hyperalgesia, by modulating miR-136/ZEB1 axis.	Shen et al., 2019
MALAT1 (metastasis-associated lung adenocarcinoma transcript 1)	Down-regulated	DRG neurons	CCI rats	Neuropathic pain behaviors such as mechanical and thermal hyperalgesia were reduced by the inhibition of MALAT1, while the loss of MALAT1 was able to depress the neuroinflammation process via the inhibition of COX-2, interleukin-1β, and interleukin-6 accompanied by miR-206/ZEB2 axis.	Chen et al., 2019
MRAK009713	Up-regulated	DRG neurons	CCI rats	MRAK009713 is a novel positive regulator of neuropathic pain in rats through regulating the expression and function of the P2 × 3 receptor.	Li et al., 2017
NEAT1 (Nuclear paraspeckle assembly transcript 1)	Up-regulated	Spinal cord	CCI rats	NEAT1 contributes to neuropathic pain development through targeting miR-381/HMGB1 axis in CCI rat models.	Xia et al., 2018
NON-RATT021972	Up-regulated	DRG neurons	Type 2 diabetes mellitus rats	NON-RATT021972 siRNA treatment suppressed the up-regulated expression and activation of the P2 × 3 receptor and reduced the hyperalgesia potentiated by the pro-inflammatory cytokine TNF-α in Type 2 diabetes mellitus rats.	Peng et al., 2017
PKIA-AS1 (PKIA antisense RNA 1)	Up-regulated	Spinal cord	Spinal nerve ligation model rats	Overexpression of PKIA-AS1 was sufficient to induce neuropathic pain-like symptoms in uninjured rats by directly regulating the expression and function of CDK6, which is essential for the initiation and maintenance of neuroinflammation and neuropathic pain.	Hu et al., 2019
SCN9A NAT (SCN9A natural antisense transcript)	Down-regulated	DRG neurons	Painful diabetic rats	SCN9A NAT correlates with the emergence of pain-related behaviors characteristic of painful diabetic neuropathy.	Li et al., 2015
T-UCRs (Transcribed ultraconserved regions)	Up-regulated	Spinal cord	Spinal nerve ligation-induced neuropathic pain in mice	T-UCR involved in the pathogenesis of neuropathic pain.	Jiang et al., 2016
uc.48+	Up-regulated	DRG neurons	Diabetic rats	The siRNA treatment of lncRNA uc.48+ may alleviate the diabetic neuropathic pain by inhibiting the excitatory transmission mediated by the P2 × 3 receptor in DRG.	Wang et al., 2016
XIST (X inactive specific transcript)	Up-regulated	Spinal cord	CCI rats	XIST accelerates neuropathic pain progression through regulation of miR-137/TNFAIP1, miR-154/STAT3, or miR-150/ZEB1 axis in CCI rat models.	Wei et al., 2018; Yan et al., 2018; Zhao Y. et al., 2018

It should be pointed out that all the current biological functions related to lncRNAs in chronic neuropathic pain are based on cellular or animal models, and research on the human body can be limited only to their expression levels, but not their biological functions (Kumar et al., 2018). The major reason is that chronic neuropathic pain is usually prone to irreversible damage in the human body, which is difficult for us to control (Gierthmuhlen and Baron, 2016). Hence, animal models of chronic neuropathic pain can be mainly grouped into four categories, including nerve injury models [such as spinal cord injury, excitotoxins, spinal hemi-section, thalamic syndrome, sciatic nerve chronic constriction injury (CCI), spinal nerve ligation, spared nerve injury, sciatic inflammatory neuritis, etc.], drug-induced chronic neuropathic pain models (such as anti-cancer-agent-induced neuropathy, vincristine-induced neuropathic pain, cisplatin-induced neuropathic pain, etc.), disease-induced neuropathy models (such as diabetes-induced neuropathy, the post-herpetic neuralgia model, human-immunodeficiency-virus-induced neuropathy, etc.), and miscellaneous models [such as chronic ethanol consumption/withdrawal-induced neuropathy, pyridoxine (vitamin B6)-induced neuropathy, inherited-induced neuropathies, etc.] (Kumar et al., 2018). Based on these models, whether the roles of lncRNAs in chronic neuropathic pain can be well reflected in the human body still needs to be further explored.

## Conclusion and Future Directions

Chronic neuropathic pain is one of the most challenging and refractory neurological diseases in a great number of patients (Gierthmuhlen and Baron, 2016). In the clinic, chronic neuropathic pain patients are often characterized by symptoms of spasticity, muscle weakness, dysesthesia, spontaneous pain, allodynia, hyperalgesia, poor proprioception, and so on (Gilron et al., 2015). Currently, in response to these symptoms of chronic neuropathic pain, there are still no clinically effective drugs for their treatment (Gilron et al., 2006). Previous studies have precisely revealed that the available analgesics, such as non-steroidal anti-inflammatory drugs and opioids, which could effectively improve the clinical signs or symptoms of nociceptive pain, did not produce an adequate effect on chronic neuropathic pain patients (Vranken, 2012). Emerging lines of evidence have also uncovered that, despite a mass of pharmacological studies performed in chronic neuropathic pain, these newly developed treatment strategies, including pharmacological, non-pharmacological, and interventional methods, remain unable to adequately alleviate the patients’ pain or reduce the pain only to a range that the patients can tolerate (Jackson, 2006). Moreover, on the other hand, epidemiological surveys have reported that the prevalence of chronic neuropathic pain in the gross population is approximately 7–10% around the world (Zimmermann, 2001; van Hecke et al., 2014). Therefore, chronic neuropathic pain represents a worldwide challenging issue that is a socio-economic burden not only for patients and caregivers but also for the healthcare system (Baron, 2009). Currently, an increasing number of clinicians believe that patients with chronic neuropathic pain have not been treated well because the pathogenesis of the disease is not completely understood (Vranken, 2012). In-depth studies of the pathogenesis of chronic neuropathic pain may provide a solid theoretical basis for the development of new drugs or agents. lncRNAs, belonging to a family of RNAs characterized as transcripts more than 200 nucleotides in length, were discovered to participate in the cellular and molecular pathological processes of multiple nervous system diseases, including chronic neuropathic pain, by regulating the expression of neuronal-function-related genes (Qureshi et al., 2010; Wu et al., 2019). Hence, many researchers have turned their attention to the study of lncRNAs, with the thought that unveiling the underlying mechanism of how lncRNAs are involved in the occurrence, development and progression of chronic neuropathic pain may offer novel avenues to prevent and treat this refractory disease (Qureshi and Mehler, 2013).

In recent years, small-molecule targeted therapies using miRNAs or lncRNAs have been carried out in many disease areas. These molecules attracted so much attention due to their biology functions in regulating protein-coding genes at different levels, including epigenetic level, transcription level, post-transcriptional level, etc. In addition, their expressions not only have temporal and spatial specificity, but also cell and tissue specificity. Thus, these biological characteristics enable drug developers to find more accurate targets when developing new drugs or agents. Nonetheless, it is undeniable that there are still many challenges in the application of small molecules in the treatment of chronic neuropathic pain, for example, the transformation between experiment research and clinical application. Given that chronic neuropathic pain can cause irreversible damage to the human body, almost all models for chronic neuropathic pain research are based on cell or animal experiments. However, there are some differences between *in vitro* experiments and *in vivo* experiments, and these differences are unpredictable. Therefore, how the results obtained by small-molecule targeted therapy in *in vitro* experiments can be transformed and applied to human body is still a big problem.

## Author Contributions

XJ and YZ proposed the concept, collected the literatures, and edited the manuscript. WW drafted the manuscript.

## Conflict of Interest

The authors declare that the research was conducted in the absence of any commercial or financial relationships that could be construed as a potential conflict of interest.
